# Role of IGF-1R in ameliorating apoptosis of GNE deficient cells

**DOI:** 10.1038/s41598-018-25510-9

**Published:** 2018-05-09

**Authors:** Reema Singh, Priyanka Chaudhary, Ranjana Arya

**Affiliations:** School of Biotechnology, Jawaharlal Nehru University, New Delhi, 110067 India

## Abstract

Sialic acids (SAs) are nine carbon acidic amino sugars, found at the outermost termini of glycoconjugates performing various physiological and pathological functions. SA synthesis is regulated by UDP-GlcNAc 2-epimerase/ManNAc kinase (GNE) that catalyzes rate limiting steps. Mutations in GNE result in rare genetic disorders, GNE myopathy and Sialuria. Recent studies indicate an alternate role of GNE in cell apoptosis and adhesion, besides SA biosynthesis. In the present study, using a HEK cell-based model for GNE myopathy, the role of Insulin-like Growth Factor Receptor (IGF-1R) as cell survival receptor protein was studied to counter the apoptotic effect of non-functional GNE. In the absence of functional GNE, IGF-1R was hyposialylated and transduced a downstream signal upon IGF-1 (IGF-1R ligand) treatment. IGF-1 induced activation of IGF-1R led to AKT (Protein Kinase B) phosphorylation that may phosphorylate BAD (BCL2 Associated Death Promoter) and its dissociation from BCL2 to prevent apoptosis. However, reduced ERK (Extracellular signal-regulated kinases) phosphorylation in GNE deficient cells after IGF-1 treatment suggests downregulation of the ERK pathway. A balance between the ERK and AKT pathways may determine the cell fate towards survival or apoptosis. Our study suggests that IGF-1R activation may rescue apoptotic cell death of GNE deficient cell lines and has potential as therapeutic target.

## Introduction

Sialic acids (SAs) are 9-carbon sugar units present at the terminal end of glycoproteins and glycolipids that regulate various cellular functions like cell proliferation, apoptosis and cell adhesion^1^. The UDP-N-acetylglucosamine 2-epimerase/N-acetylmannosamine kinase (GNE) is a 79-kDa key bifunctional enzyme of sialic acid biosynthesis that consists of N-terminal epimerase and C-terminal kinase domains^2^. Mutations in GNE lead to the neuromuscular disorder, GNE myopathy, characterized by muscle weakness and atrophy, with protein aggregation seen in muscle biopsy samples^3^. Biallelic mutations of this gene have been reported in GNE myopathy patients worldwide. Most patients eventually become wheel chair bound within 15 years of disease onset. There are more than 180 different GNE mutations known^4^. However, due to (i) rareness of the disease (unfamiliar to most neurologists), (ii) non-inclusion of GNE genetic testing for neurological indications, and (iii) non-specific symptoms at disease onset (foot-drop, balance impairment), the diagnosis of these patients is difficult and often delayed. Also, there is no effective treatment of the disease. Current human clinical trials are based on substrate supplementation with the SA or its precursor N-acetylmannosamine (ManNAc, NIH, USA)^5^. Single doses of oral ManNAc administration in GNE myopathy patients resulted in increased plasma free SA levels indicating restoration of intracellular SA synthesis^6^. Supplementation with SA delivered by aceneuramic acid extended release stabilized muscle strength in GNE myopathy patients^7^, however, this study did not demonstrate a statistically significant difference in the muscle strength of patients compared to placebo and hence, discontinued from clinical trials (Ultragenyx, 2017).

A major limitation in drug development for GNE myopathy is that the exact pathophysiology of the disease is not well-understood. Apart from SA deficiency, other theories for the pathophysiology of GNE myopathy have been suggested, including a role of the GNE protein in regulating other cell functions. Beside its fundamental function in SA biosynthesis, GNE was suggested to regulate sialyltransferase mRNA levels, affecting cell proliferation^8^. GNE was shown to interact with α-actinin1/2, CRMP-1 (collapsing response mediator protein-1) and PLZF (Promyelocytic leukemia zinc finger protein) with unclear functions^9–11^. Upregulation of molecular chaperones such as GRP78, GRP94, Calreticulin and cell stress molecules such as β-crystallin and iNOS was also observed in GNE Myopathy^12–14^. Recently, GNE has been shown to affect cell adhesion via hyposialylation of cell surface β-1 integrin^15^. Further, domain specific mutation effects of GNE were observed on mitochondrial dependent cell apoptosis^16^. These studies indicate that GNE may play alternate roles in regulating cellular functions.

The majority of cell surface receptors are glycosylated/sialylated, and hyposialylation of muscle glycoproteins is thought to be responsible for muscle deterioration in mouse models of GNE myopathy^17–20^. Among various cell surface receptors, IGF-1R (insulin-like growth factor receptor) expression is important for myoblast proliferation and maintenance of normal muscle mass^21–26^. In humans, a homozygous partial deletion of IGF-1R plays a neuroprotective role in various neurodegenerative disorders including Alzheimer’s and Huntington disease^27,28^. Importantly, proper glycosylation of IGF-1R is crucial for its function^29^. Desialylation of IGF-1R by neuraminidase I causes quenching of proliferative response in L6 myoblasts^30^. However, the exact functional significance of aberrantly glycosylated IGF-1R is not clear. How the pattern and magnitude of glycosylation/sialylation affects its neuroprotective role is not yet understood.

Structurally, IGF-1R consists of two subunits: α and β. The extracellular α subunit (130 KDa) is sialylated via α 2, 6-linkages and binds the IGF-1R ligands (IGF-1, IGF-2), while the intracellular β subunit (97 kDa) transmits a downstream signal by autophosphorylating tyrosine residues. IGF-1R and its natural ligands regulate multiple cellular functions such as protection of cells from oxidative stress^26,31^ and apoptosis to promote cell survival^32^. Alterations in IGF-1R downstream signaling molecules such as PI3K/AKT are reported in various cancers^33,34^. Additionally, induction of IGF-1R with IGF-1 and its signaling components provides endogenous neuroprotection and repair in a brain injury mouse model^35^. Muscle cell growth, proliferation and inhibition of apoptosis is associated with increased expression of IGF-1R and αVβ3 integrin receptors^36^. Therefore, it would be of interest to explore the effect of IGF-1-induced IGF-1R signaling in hyposialylated GNE-deficient cells.

In the present study, we aim to investigate the role of IGF-1R in cell regulation of GNE deficient cells. We hypothesize that an IGF-1R ligand such as IGF-1 could elicit a cell survival response in apoptotic GNE deficient cells. To address this, we have used a HEK293 cell-based system where GNE is either knocked down or the cells over-express mutant GNE recombinant protein. These cell lines were earlier established to study cellular response to reduced SA content and GNE enzyme activity^15,16^. Our study indicates the importance of IGF-1R in maintaining a balance between cell survival and apoptosis of GNE deficient cells that are of interest to be explored further for drug design in disorders associated with sialic acid deficiency.

## Results

### Mitochondrial defects in GNE deficient cells

**Figure 1:**
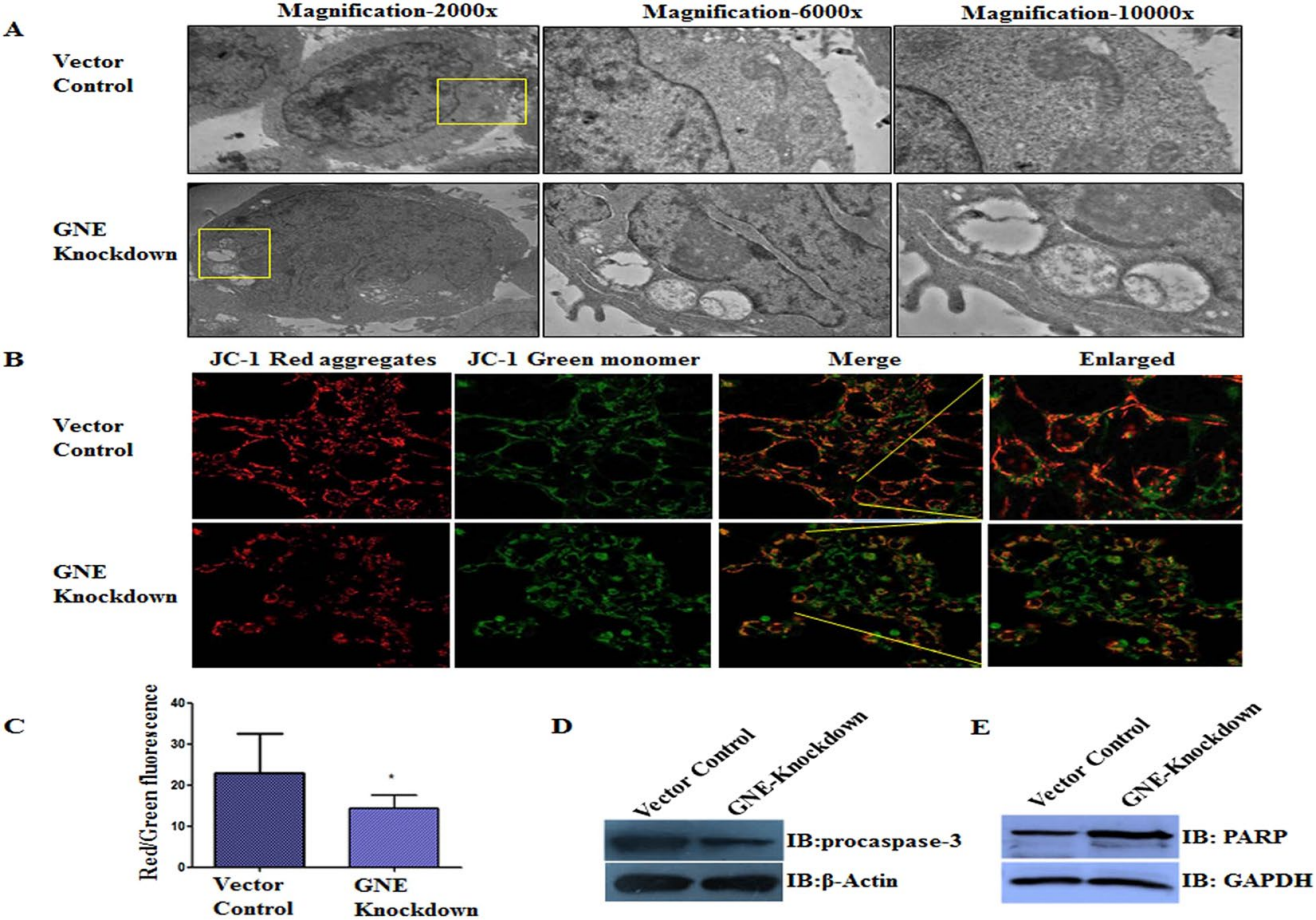
Effect of GNE knockdown on mitochondrial dysfunction: (**A**) Morpho-structural analysis of mitochondria: representative TEM image showing alterations in mitochondrial morphology of GNE knockdown cells, compared to vector control cells. (**B**) Effect of GNE knockdown on dissipation of mitochondrial membrane potential (as measured by JC-1) using confocal microscopy, magnification 60×. Loss in mitochondrial membrane potential or depolarization is indicated by increased presence of JC-1 as monomeric form (green) and consequently leading to a decrease in the red/green fluorescence intensity ratio. (**C**) Histogram shows the ratio of red to green fluorescence intensity observed in GNE knockdown cell lines characterizing mitochondrial membrane potential. (**D**) Immunoblot analysis of cell lysates from indicated cell lines with anti-procaspase 3, β-actin was used as loading control. (**E**) Immunoblot analysis of cell lysates from indicated cell lines with anti-PARP antibody, GAPDH was used as loading control. The statistical significance was assessed by one-way ANOVA test. P values for * is <0.05.

Our previous reports indicated that mutations in GNE disrupted mitochondrial structure and function leading to cell apoptosis^16^. Different GNE mutations had varied effects on the extent of cellular damage which may be attributed to altered interactions of mutated GNE polypeptides with other effector molecules. In order to identify such interactions and potential drug targets, we extended our study to cell surface receptor molecules which are sialylated and may respond differently due to altered sialylation. One of the key sialylated receptors present on the cell surface whose role in proliferation, cell survival and apoptosis is well established include IGF-1R^37–39^. We studied the effect of GNE deficiency on IGF-1R activity in HEK 293 cells stably transfected with shRNA against endogenous GNE. This inhibition of endogenous GNE resulted in 70% reduction in GNE expression and 80% ± 2 reduction in total cellular sialylation^15^. We estimated the extent of mitochondrial damage in these cells via transmission electron microscopy (TEM) (Fig. 1A). The TEM images showed swollen and vacuolar mitochondria in GNE knockdown cells compared to vector control cells. The depolarization of mitochondrial membranes was assessed quantitatively using JC-1 dye. The green monomer JC-1 cyanine dye (5,5′,6,6′-tetrachloro-1,1′,3,3′-tetraethylimidacarbocyanine iodide) forms red aggregates in energized mitochondria^40^. However, when mitochondrial membranes are depolarized, the dye fails to form red aggregates. We observed higher green fluorescence of JC-1 compared to red aggregated form in GNE knockdown cells, indicative of mitochondrial membrane depolarization (Fig. 1B). Hence, the ratio of red to green fluorescence in mitochondrial membranes of GNE knockdown cells was significantly reduced compared to vector control (Fig. 1C). This study supported our previous report on morpho-structural changes of mitochondria in absence of functional GNE^16^. Further, the downstream effector molecules of mitochondrial-dependent apoptotic pathways were found to be activated such as levels of procaspase-3^41^ were reduced suggesting its cleavage into active caspases (Fig. 1D). Also levels of cleaved PARP (PolyADP ribose polymerase)^42^ were detected with the appearance of a 90 kDa lower band as opposed to 113 kDa uncleaved PARP in GNE knockdown cells indicative of DNA damage (Fig. 1E)^43^. Our study supports and confirms the occurrence of mitochondrial dependent apoptosis in cells with incompetent GNE.

### GNE inhibition results in hyposialylation of IGF-1R

**Figure 2:**
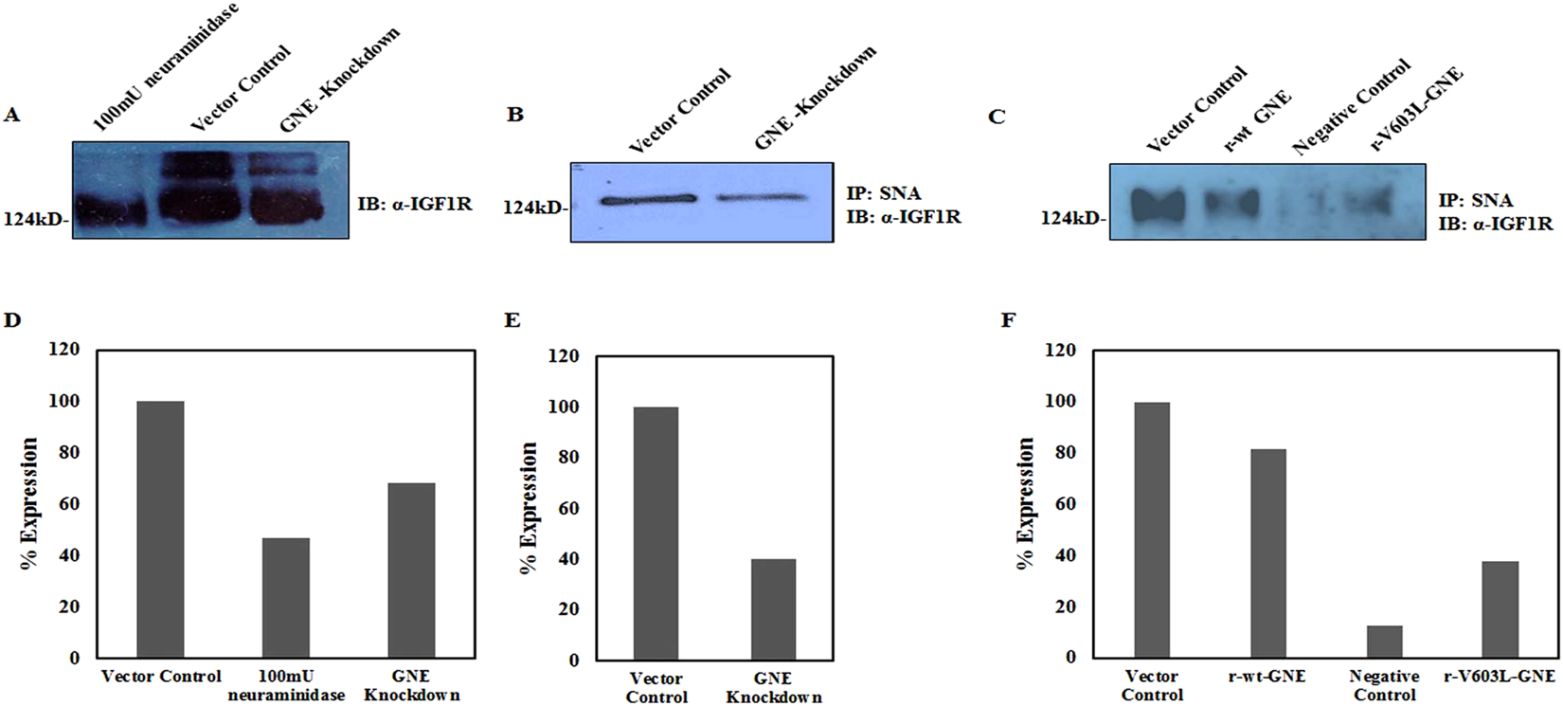
Determination of IGF-1R sialylation levels using Lectin affinity assay: (**A**) Sialylation status of IGF-1R: Equal amount of cell lysates from vector control cells were treated with 100 mU of *C. perfringen*s sialidase and untreated lysates from GNE knockdown cells were separated on 8% SDS-PAGE followed by immunoblotting with anti-α IGF-1R. (**B**) SNA Lectin affinity assay: Sialylated IGF-1R from GNE knockdown cells and scrambled shRNA vector control cell was pulled down using biotin labelled SNA/streptavidin-coupled agarose and immunoblotted with anti-α IGF-1R. (**C**) Sialylated IGF-1R complexes from GNE mutant and pcDNA3 transfected vector control cells were pulled down using biotin labelled SNA/streptavidin-coupled agarose and immunoblotted with anti-α IGF-1R. Sialylated IGF-1R complexes from 100 mU neuraminidase treated vector control cells that were pulled down using biotin labelled SNA/streptavidin-coupled agarose served as negative control in this experiment. Equal amount of protein was used as input lysates. (**D**–**F**) are showing representative densitometry graphs of (**A**–**C**), respectively, normalized to vector control.

Since the key function of GNE is epimerization and phosphorylation of precursors in the sialic acid biosynthetic pathway, absence of functional GNE would cause hyposialylation and affect sialylation of cell surface receptors^15,30^. With an aim to understand how decreased GNE expression might affect sialylation and function of IGF-1R, we first determined sialylation levels of IGF-1R in GNE knockdown cells by immunoblotting. The SDS-PAGE migration of α-IGF-1R from GNE knockdown cell extracts was altered, and was similar to neuraminidase *(C. perfringens*) treated vector control extracts, indicating reduced sialylation of IGF-1R in GNE knockdown cells (Fig. 2A). This was further confirmed by immunoprecipitation with *Sambucus nigra* agglutinin (SNA) lectin, which predominantly binds sialic acid residues specific to terminal galactose in α-2, 6 (and some extent to α-2, 3) linkages^44^. Approximately 40% reduction in IGF-1R sialylation from GNE knockdown cells was observed compared to vector control (Fig. 2B). Further, approximately 55% reduction in IGF-1R sialylation was observed in cells with mutation in GNE kinase domain (V603L) (Fig. 2C). Densitometry analysis of each blot is shown in Fig. 2D–F, respectively. These results indicate that reduced GNE enzyme activity leads to hyposialylation of IGF-1R.

### Effect of hyposialylation on IGF-1R localization

**Figure 3:**
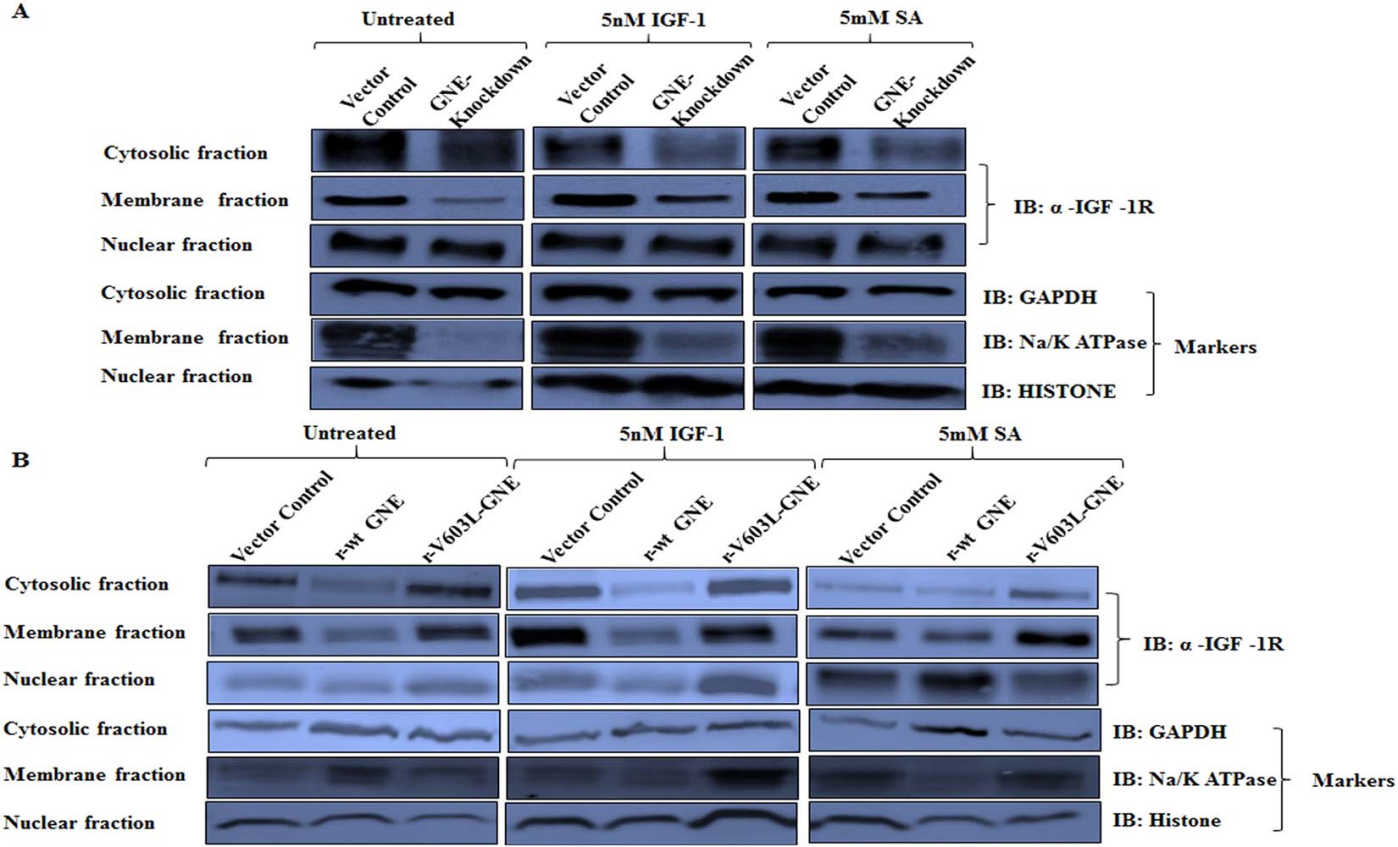
Localization of IGF-1R by sub-cellular fractionation: Membrane, nuclear and cytoplasmic fractions from different cell lines were obtained as described in Material & Methods. (**A**) Distribution of IGF-1R in various fractions of GNE knockdown cells and (**B**) GNE mutant cells is shown compared to vector control. The cells were untreated, IGF-1 treated or Sialic acid (5 mM SA) supplemented as described in Methods. Immunoblotting was done with anti-α IGF-1R antibody, anti-GAPDH (cytosolic marker), anti-Na/K ATPase (membrane marker) and anti-Histone H3 (nuclear marker).

IGF-1R is reported to localize in the plasma membrane, cytosol and nucleus of cells^45^. Absence of N-linked glycosylation of IGF-1R affects its membranous localization^46^. To understand the effect of hyposialylation on IGF-1R localization, we determined the expression levels of IGF-1R in cytosol, membrane and nuclear fractions of GNE knockdown and mutant cells. As shown in Fig. 3, IGF-1R was distributed in cytosol, membrane and nuclear fractions of all the cell lysates. In GNE knockdown cells, reduced IGF-1R levels were observed in membrane fraction compared to cytosolic fraction (Fig. 3A). After treatment with IGF-1, IGF-1R levels increased in membrane fraction of all the cell lysates compared to cytosolic fractions indicating IGF-1R migration to the membrane (Fig. 3A,B). Nuclear localization of IGF-1R was unaltered in GNE knockdown cells while marginal increase in nuclear retention of IGF-1R was observed in V603L GNE mutant cells. Supplementation with sialic acid showed restoration of IGF-1R distribution in GNE deficient cell lines similar to vector control. These experiments were repeated independently three times. The densitometry analysis of immunoblots depicting ratio of IGF-1R distribution in membrane versus cytoplasm is shown in Table 1. The ratio of membrane versus cytosolic IGF-1R localization in the untreated panel indicates cytosolic retention of hyposialylated IGF-1R in GNE knockdown cells but not in V603L GNE mutant cells. However, after IGF-1 treatment, increased IGF-1R membrane distribution indicates receptor mobilization from the cytosolic fraction in all the cell lines. This suggests that hyposialylation did not affect distribution/migration of IGF-1R after IGF-1 treatment.

**Table 1:** Ratio of IGF-1R distribution in various sub-cellular fractions: Ratio of IGF-1R levels in membrane versus cytosolic fraction of various cell lines as calculated by densitometry analysis of Fig. 3 blots.

Sample	Membrane *versus* Cytosolic			Membrane fraction
	Untreated	5 nM IGF-1	5 mM SA	Ratio of IGF-1 treated versus Untreated
**GNE Knockdown Cells**				
Vector Control	1.29	1.73	1.2	1.55
GNE Knockdown	0.75	0.93	1.77	1.25
**GNE Mutant Cells**				
Vector Control	1.09	1.42	4.26	2.32
r- WT GNE	1.26	1.59	4.45	2.17
r-V603L GNE	1.24	1.58	3.44	1.96

Comparison determines IGF-1R distribution with respect to GNE deficiency. The last column indicate the ratio of IGF-1R distribution in membrane fraction after IGF-1 treatment versus untreated to determine plasma membrane migration pattern.

### Effect of altered IGF-1R sialylation on its downstream molecules/pathways

**Figure 4:**
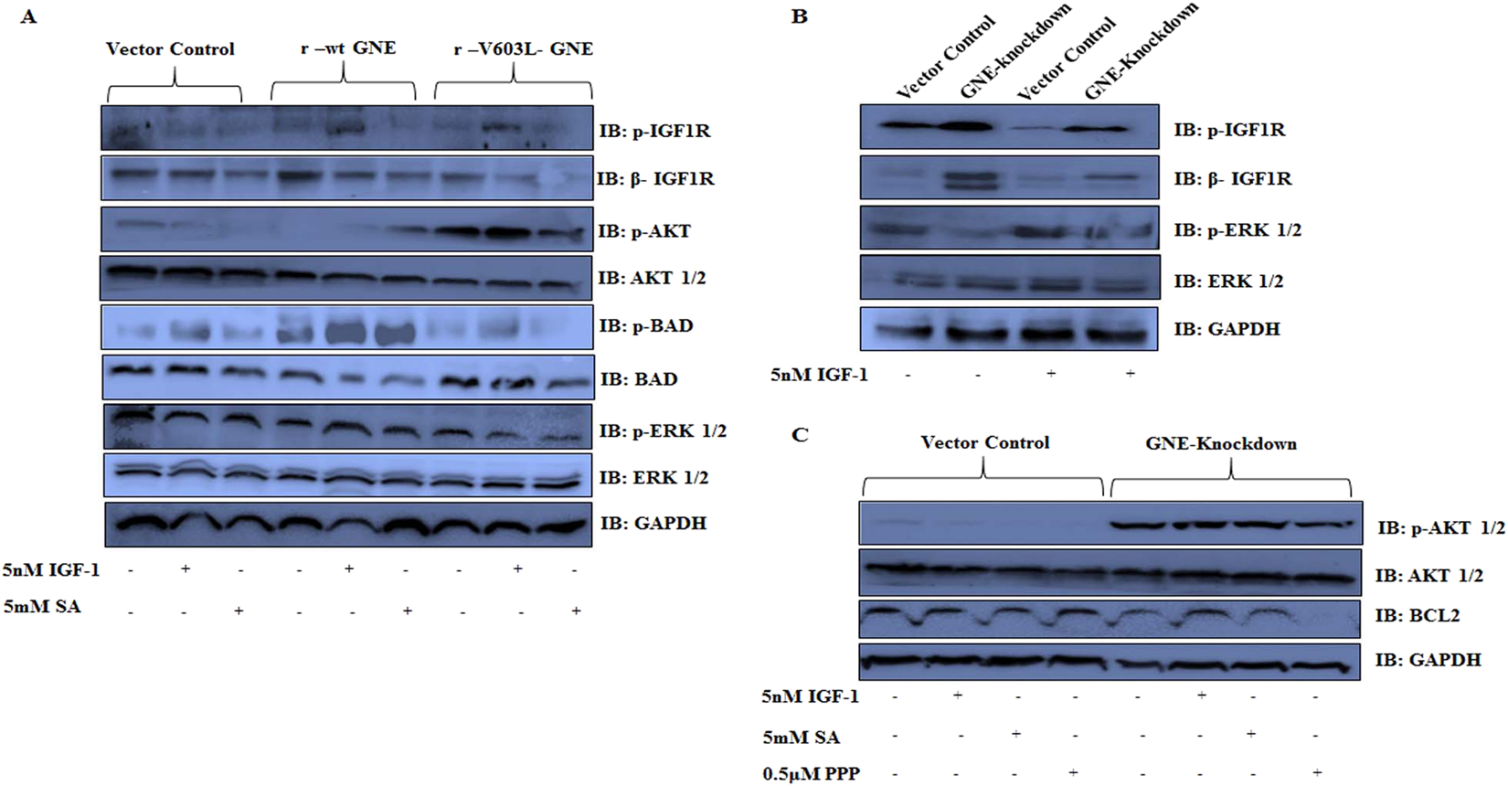
Effect of GNE deficiency on IGF-1R downstream signaling pathway: Cells were grown in DCCM, followed by treatment with 5 nM IGF-1 for 10 min or treatment with 5 mM Sialic Acid or 0.5 μM PPP (IGF-1R inhibitor) for 24 h. (**A**) GNE mutant cells along with r-wt GNE and vector control cell lysates after treatment with or without 5 nM IGF-1 or 5 mM SA were separated on SDS–PAGE and immunoblotted with p-IGF-1R/β-IGF-1R, p-AKT/AKT, BAD/p-BAD, p-ERK/ERK1/2 and GAPDH antibodies. (**B**) GNE knockdown and vector control cell lysates with or without 5 nM IGF-1 treatment were separated on SDS–PAGE and immunoblotted with p-IGF-1R/β-IGF-1R, p- p-ERK/ERK1/2 and GAPDH antibodies. (**C**) GNE knockdown and vector control cell lysates after treatment with or without 5 nM IGF-1, 5 mM SA or 0.5 μM PPP (IGF-1R inhibitor) were separated on SDS–PAGE and immunoblotted with p-AKT/AKT, BCL2 and GAPDH antibodies.

Since sialylation of the IGF-1R α subunit via α 2, 6 linkages is important for its conformation and ligand binding^47^, it is of interest to determine whether hyposialylation of IGF-1R affects its downstream signaling events. IGF-1R activation after ligand binding activates AKT kinases/pathway linked to prevention of apoptosis and the ERK pathway associated with growth and proliferation. Both these signaling pathways synergize to result in the prevention of apoptosis and stimulation of cell growth^48^. Both AKT and ERK activation cause phosphorylation of BAD (BCL2-associated death promoter)^49^ so that BAD dissociates from BCL2 (B cell lymphoma 2) and binds 14-3-3 protein. This release of BCL2 supports anti-apoptotic activity and cell survival. In our study, IGF-1R phosphorylation was observed in GNE knockdown and mutant cells indicating activation of IGF-1R after IGF-1 treatment (Fig. 4A,B). Activated IGF-1R transduced the signal^49,50^ to AKT as increased levels of pAKT were observed in GNE knockdown and mutant cells (Fig. 4A,C). AKT activation led to increased BAD phosphorylation in IGF-1 treated GNE mutant cell lines that supports cell survival (Fig. 4A). On the other hand, ERK showed reduced phosphorylation in GNE mutant cells even after IGF-1 treatment suggesting downregulation of the ERK pathway. Previous reports also suggest downregulation of ERK in GNE deficient cells^8,15,48^. The study was also performed in GNE knockdown cells with similar observations. Phosphorylation of AKT was increased in GNE knockdown cells after IGF-1 treatment while ERK phosphorylation was reduced. In addition, BCL2 levels were increased indicative of anti-apoptotic response in these cells after IGF-1 treatment (Fig. 4B,C). Thus a possible balance between cell survival and apoptotic pathway in GNE deficient cells may govern the fate of the cell.

The specificity of the response was determined by treatment with an IGF-1R specific inhibitor, Picropodophyllin (PPP) that showed reduced BCL2 levels in GNE knockdown cells indicative of apoptotic response (Fig. 4C). In addition, SA supplementation did not cause AKT and BAD phosphorylation in GNE mutant cells, indicating that sialic acid alone was not sufficient to rescue the apoptotic response (Fig. 4C). This is consistent with our earlier reports where the mitochondrial damage due to GNE mutation could not be reverted by sialic acid supplementation alone^16^. Our study indicates suppression of pro-apoptotic and activation of anti-apoptotic pathway in GNE deficient cells after treatment with IGF-1.

### Treatment with IGF-1 rescues cell apoptosis

**Figure 5:**
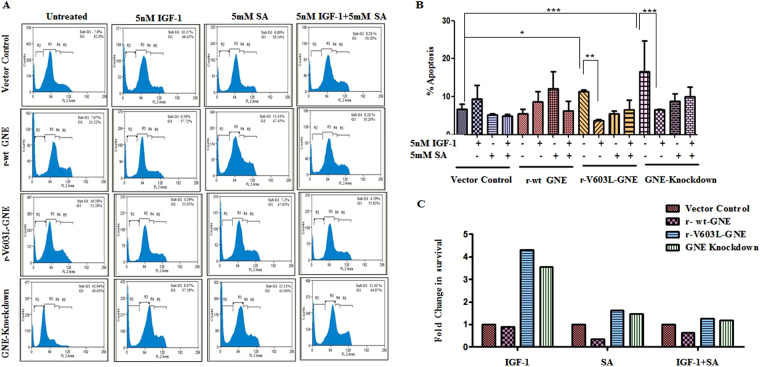
Cell cycle analysis of GNE deficient cells: The cell cycle analysis using propidium iodide was done by Flow Cytometry. (**A**) Representative histogram of the distribution of GNE deficient cells and GNE mutant cells in various phases of the cell cycle. Cells were treated with 5 nM IGF-1 alone, Sialic acid alone or a combination of both (IGF-1 + SA) (**B**) Graphical representation of cells in sub G0/G1 phase (% apoptosis).The statistical significance was assessed by One-way ANOVA test followed by Bonferroni post tests, P values for *, ** and *** are <0.05, <0.001 and <0.0001, respectively. (**C**) Graphical representation showing fold change in cell survival which is calculated by taking the ratio of percentage sub G_0_/G_1_ population cells (represented by R2 in histograms 5A) obtained from 5B of IGF-1 treated versus untreated, SA treated versus untreated and IGF-1 + SA treated versus untreated, normalized to vector control.

**Figure 6:**
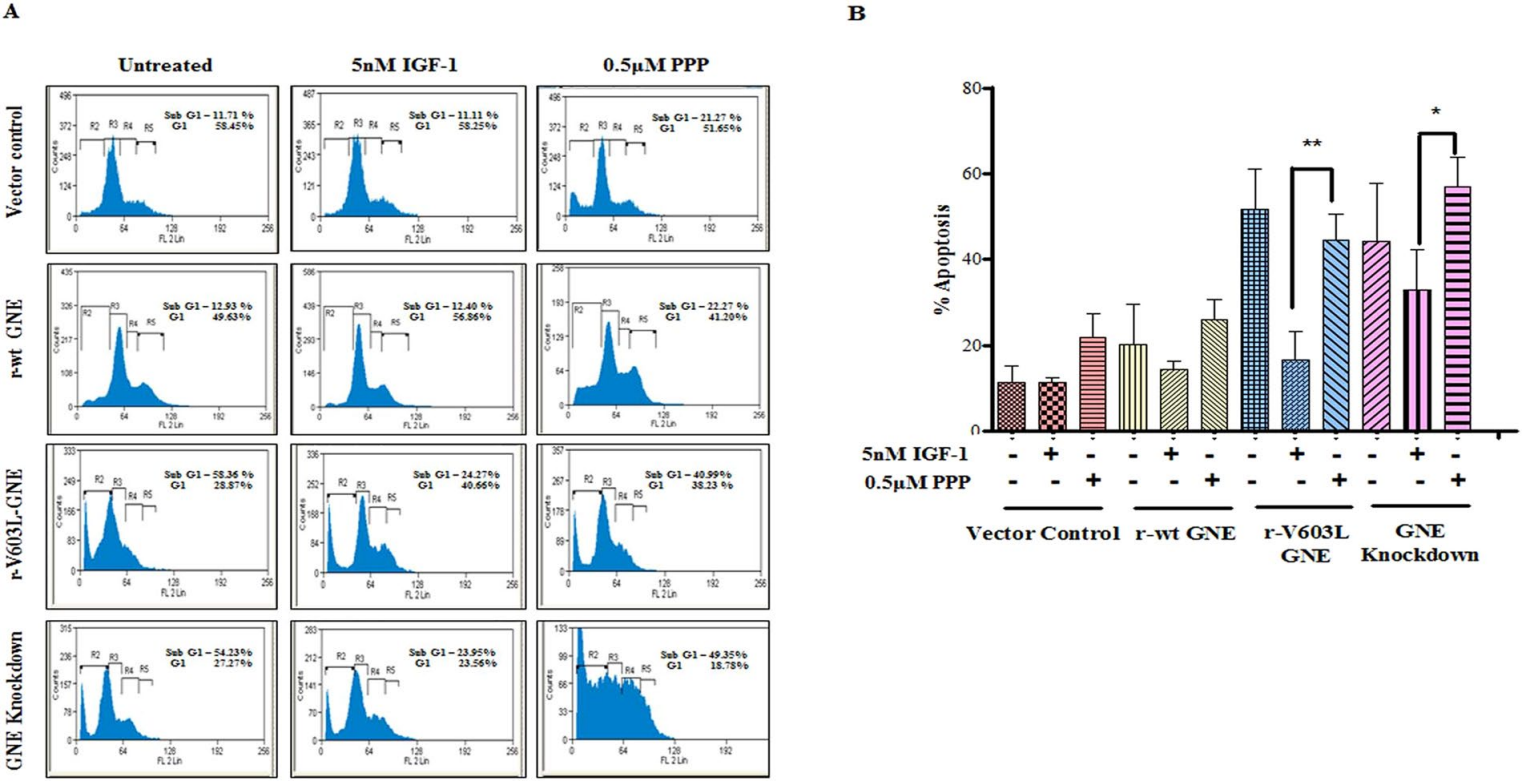
Cell cycle analysis of GNE deficient cells in the presence of IGF-1R inhibitor Picropodophyllin (PPP): The cell cycle analysis using propidium iodide was done by Flow Cytometry. (**A**) Representative histogram of the distribution of GNE knockdown cells, GNE mutant cells, wild type GNE and vector control cells in various phases of cell cycle where sub G_1_ population represent apoptotic cells. Cells were treated with 5 nM IGF-1 and 0.5 μM PPP. (**B**) Graphical representation of cells in sub G_0_/G_1_ phase (% apoptosis). The statistical significance was assessed by one-way ANOVA test followed by Bonferroni post tests. P values for * and ** are <0.05 and 0.001 respectively.

The effect of hyposialylation on IGF-1R function was further assessed by understanding cell cycle progression of GNE knockdown and mutant cells. Cell cycle analysis using propidium iodide by Flow Cytometry measures cellular DNA content and reveals distribution of cells in three major phases of the cell cycle (G0/G_1_ versus S versus G_2_/M)^51^. Apoptotic cells with fragmented DNA constitute sub-G1 population. A representative histogram of the distribution of GNE deficient cells and GNE mutant cells in various phases of the cell cycle is depicted in Fig. 5A. The sub G1 population of cells is represented by R2 in Fig. 5A. A shift in the R2 region was observed in GNE mutant or knockdown cells compared to vector control. Almost 11.4% ± 0.26 and 16.5% ± 4 apoptotic cells were observed in sub G0/G1 (R2) population of GNE mutant and knockdown cells, respectively, compared to vector control (6.7% ± 0.76) or r-wt-GNE (5.5% ± 0.69) (Fig. 5B). This indicates that a significant number of cells undergo apoptosis in absence of functional GNE. Interestingly, after treatment with IGF-1 only 3.7% ± 0.12 and 6.5% ± 0.08 GNE mutant and knockdown cells, respectively, were observed in sub G0/G1 phase (Fig. 5B). This indicates a significant recovery of apoptotic cells towards cell survival after IGF-1 treatment. SA supplementation alone resulted in 8.1% ± 0.66 apoptosis in GNE mutant cells and 13% ± 1.3 apoptosis in GNE knock down cells, respectively, suggesting insignificant recovery of apoptosis in GNE deficient cells compared to untreated cells. Surprisingly, SA along with IGF-1 showed 9.1% ± 0.71 apoptosis in GNE mutant cells and 13% ± 2.3 apoptosis in GNE knockdown cells. All these experiments were performed in triplicates and data compiled from three independent experiments. Quantitatively, a 4.3 fold increase in cell survival was observed in GNE mutant cells and a 3.56 fold increase in cell survival of GNE knockdown cells was observed after IGF-1 treatment (Fig. 5C). SA supplementation alone or in combination with IGF-1 showed only a 1.4 fold increase in cell survival of GNE deficient cells. To further support the above observations, the experiments were performed in the presence of Picropodophyllin (PPP, IGF-1R inhibitor). As shown in Fig. 6(A,B), 44% apoptotic cells were observed in the R2 region of GNE mutant and 57% in the R2 region of GNE knockdown cells suggesting that inhibition of IGF-1R led to cell cycle arrest in GNE deficient cells. Our study indicates that IGF-1 specific treatment of GNE deficient cells may cause cells to survive and overcome apoptosis.

Future studies on other cellular mediators of the pathway would unravel the involvement of IGF-1R in apoptosis/survival associated with deficiency of GNE and/or a non-functional SA biosynthetic pathway.

## Discussion

**Figure 7:**
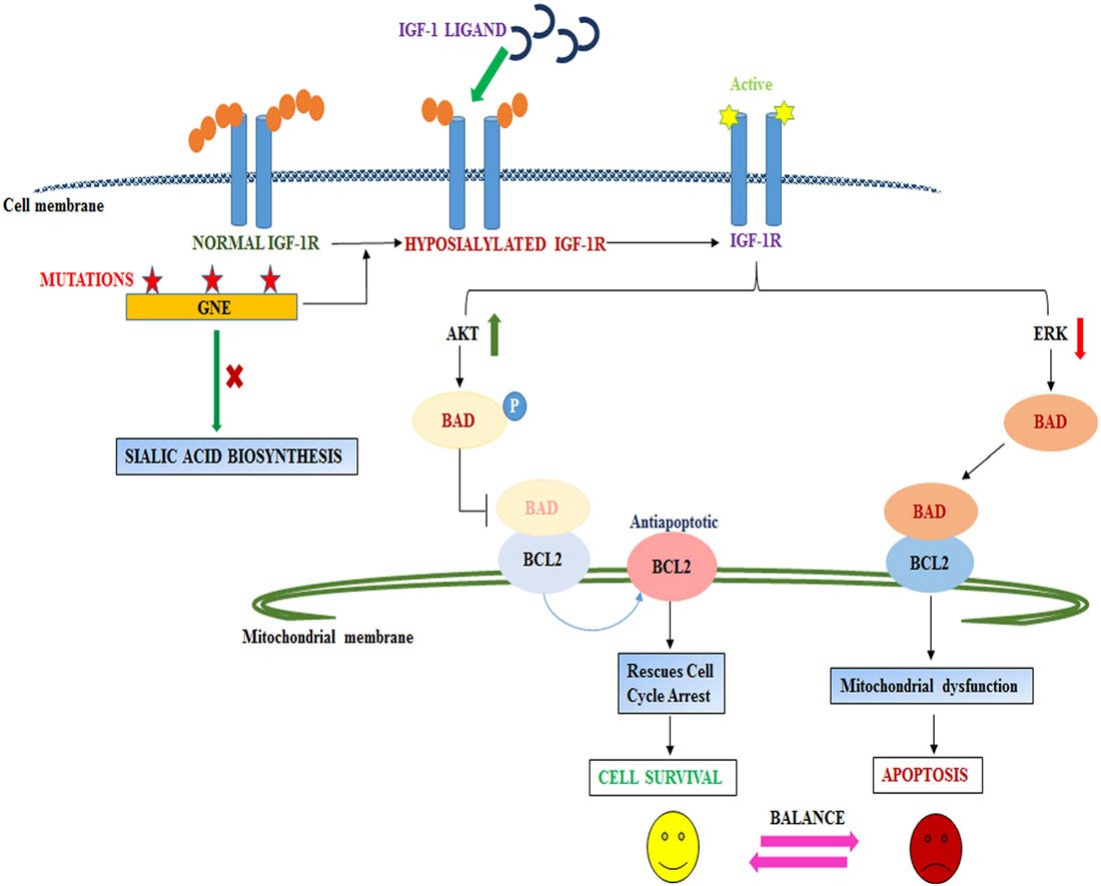
Proposed Model: The figure shows that mutation in GNE inhibits sialic acid synthesis that may cause hyposialylation of IGF-1R. IGF-1R gets activated upon IGF-1 ligand binding and transduces the downstream signal to activate AKT that phosphorylate BAD leading to its dissociation from BCL2 which is anti-apoptotic signal to rescue cell cycle arrest and cell happily survives. Simultaneously, ERK may be downregulated in hyposialylated cells to cause dephosphorylation of BAD and its association with BCL2 continues to mediate mitochondrial dysfunction and cell apoptosis. A balance between cell survival and apoptosis pathway will determine the cell fate.

Studies towards understanding patho-mechanism of GNE myopathy at molecular and cellular level indicate disturbance in apoptotic signaling, stress response, cytoskeletal architecture and mitochondrial deregulation^9^. Clinically, patients with GNE myopathy exhibit loss of muscle mass. Our previous report showed mitochondrial deregulation leading to apoptosis in cell culture lines overexpressing mutant GNE^16^. Another study proposed increased amyloid β-peptide endocytosis to be the cause of apoptosis in GNE myopathy^52^. However, there is a balance between survival and apoptosis that determines cell fate. Several cell survival mechanisms might get activated in order to rescue cell death. In the present study, one such survival–apoptosis associated signaling mechanism is deciphered through effect of IGF-1R hyposialylation in GNE deficient cell lines (Fig. 7).

In our previous study, we found that GNE mutation-specific effects caused varied extent of cell apoptosis through mitochondrial dysfunction^16^. The aim of the present study is to understand survival mechanisms countering apoptosis in GNE-deficient cells. Since PI3K/AKT, IGF-1 and ERK signaling networks are implicated in cell growth, proliferation, survival and metabolism, we focused our studies on the IGF-1R regulated signal transduction cascade.

Similar to our previous work, we have established and validated apoptosis in GNE knockdown cells via mitochondrial structural and functional disruption leading to nuclear/DNA damage or apoptosis. Vacuolar mitochondria with disrupted cristae and mitochondrial membrane depolarization, caspase activation and PARP cleavage was observed in GNE knockdown cells. Other studies also suggested mitochondrial deregulation at transcriptome and morphological level in absence of functional GNE^53^. Our study indicates that mitochondrial metabolism might be a primary event contributing to cellular dysfunction in GNE deficient cells.

Defects in GNE predominantly cause hyposialylation of glycoconjugates. Since the role of glycosylated IGF-1R in proliferation, cell survival and apoptosis is well established and desialylation disrupts its function in muscle cells, we chose to study the effect of GNE knockdown on IGF-1R function^30,54^. We showed decreased sialylation of α IGF-1R in mutant rGNE–V603L and GNE knockdown cells with reference to vector control using SNA lectin affinity assay that specifically binds terminal sialic acid residues. This is in concordance with our previous reports that glycosylated receptor molecules such as integrins get hyposialylated in absence of functional GNE^15^. Since GNE myopathy is characterized by adult onset of disease, it raises the possibility of hormone regulated control. In fact, IGF-1R glycosylation is essential for hormone dependent activation, and desialylation of IGF-1R could contribute to its functional defect^54,55^.

Previous reports have shown that aberrant glycosylation of IGF-1R prevented receptor translocation to the plasma membrane^54,56^. The subcellular fractionation studies showed preferential retention of IGF-1R in the cytosol in GNE knockdown cells while IGF-1R appeared to migrate to the plasma membrane after IGF-1 treatment. Nuclear localization of IGF-1R and insulin receptor substrates IRS-1, 2, 3 has been reported in response to oncogenic stimuli leading to altered gene regulation and expression^46,57–59^. Similar findings were reported for PECAM (platelet endothelial cell adhesion molecule), which contains α-2, 6 sialylation^60^. Our study supports the notion that glycan based methods can be exploited for modulation of angiogenesis and age related disorders such as GNE myopathy.

Sialylated cell surface receptors interact with ligand through extracellular domain and sialylation status affects receptor dimerization as well as ligand binding such as for EGFR (epidermal growth factor receptor)^61^. We observed IGF-1 specific response of IGF-1R that led to increased phosphorylation and activation of the receptor suggesting IGF-1 could bind the hyposialylated IGF-1R and dimerize the receptor for transduction of downstream signal. The intracellular IGF-1R migrated to membrane in presence of IGF-1 indicating receptor mobilization. The downstream signal was transduced leading to AKT activation that eventually rescued the apoptotic cell death of GNE deficient cells as evident by reduced number of cells in sub G0/G1 phase of cell cycle. Increased AKT phosphorylation and activation has been reported in cultured myoblasts of GNE myopathy patients^62^. IGF-1 supplementation improved cell survival rates to almost 50% above SA supplemented cells. Addition of IGF-1 to SA supplemented cells also increased the cell survival response marginally suggesting IGF-1 may turn the apoptotic cell switch towards survival. Since we also observed BAD and ERK dephosphorylation of GNE deficient cells after IGF-1 induction, there seem to be a striking balance between apoptosis and survival that determines cell fate. It would be of interest to study the regulator of switch or threshold that drives cell towards disease phenotype eventually causing accumulation of aggregated proteins.

An unanswered question remains as to how hyposialylated IGF-1R interacts with IGF-1 and induces the signaling response. It is possible that IGF-1 independent activation of IGF-1R by other kinases such as FAK and integrin 1β kinase might be responsible for its activation even in absence of GNE^63^. It is also possible that mechanisms modulating the rate of SA synthesis and thus, sialylation of IGF-1R are critical for IGF-1R signaling response. Besides, there is still a need to prove whether GNE deficient cells contain functionally active intracellular IGF-1R and whether intracellular or extracellular IGF-1 pathway is affected in the absence of proper glycosylation leading to cell apoptosis. Our cell cycle analysis study showed considerable decline of GNE deficient cells in sub G1 phase after IGF-1 treatment. IGF-1 is a potent neurotrophic factor that has neuroprotective properties in the central and peripheral nervous systems^64,65^. IGF-1 has also been reported as trait, progression and prediction marker in Parkinson’s disease^65^. In addition, IGF-1 (because of its ability in improving flow metabolism) could be explored as a new cardiovascular disease treatment option for diseases like ischemic heart disease and heart failure^66^. IGF-1 was also found to be helpful in spinal and bulbar muscular atrophy (SBMA), or Kennedy disease, in which IGF-1 decreased the aggregation of androgen receptor within muscles and overexpressing IGF-1 inside muscles protected motor neurons in the central nervous system^67^. Rescue of apoptosis using such treatments is an interesting observation which can be exploited as an alternate route for therapy in future. However, using HEK cell based system has a limitation and the study needs to be validated in muscle cells. Detailed *in vivo* studies in specific mouse models will entail future prospects for exploring these cell surface receptor molecules as drug targets for GNE Myopathy.

## Materials and Methods

### Cell culture

HEK293 cells with endogenous *GNE* knockdown, stably overexpressing wild type GNE (wtGNE) and r-V603L GNE mutant were used for the study^15^. GNE inhibition in knockdown cell lines was confirmed with 70 ± 5% reduction in GNE protein expression by immunoblotting with anti-GNE antibody (Santa Cruz Biotechnology, USA), reduction in GNE enzyme activity and sialic acid content^15,16^.

### Transmission Electron Microscopy

1 × 10^7^ cells were fixed in 2.5% glutaraldehyde (pH 7.2) followed by secondary fixation in1% osmium tetra oxide solution. After dehydration in a graded series of ethanol (50%, 70%, 90% and 100%), the cells were embedded in epoxy resin. Thin sections (70 nm) were cut using an ultramicrotome. The sections on the grids were stained with uranyl acetate and lead citrate. The sections were examined on a Transmission Electron Microscope (*JEOL 2100F*) in Advanced Instrument Research Facility, JNU.

### Mitochondrial membrane potential

1 × 10^5^ cells of GNE knockdown or empty vector control cells were seeded in glass bottom dishes for 24 h in DCCM medium. Then, cells were incubated with JC-1 Dye (Invitrogen, USA) at a concentration 2.5 μg/ml for 10–20 min. The fluorescence was detected using confocal microscopy (Nikon TM) at 60× magnification with oil immersion objective and red/green florescence ratio was analyzed with the help of NIS-Elements Viewer 3.0 software.

### Lectin Affinity Assay

600 μg of cell lysate was incubated for 3 h at 4 °C with 6 μg biotinylated *Sambucus nigra* lectin (SNA). 20 μl of streptavidin-agarose beads (Sigma, USA) were added and then incubated for 4 h at 4 °C with rotation. Lectin/glycoprotein complexes were collected by brief centrifugation and washed three times with lysis buffer, followed by one wash with 1× PBS. After brief centrifugation, precipitated proteins were released from bead complexes by boiling in sodium dodecyl sulfate-polyacrylamide gel electrophoresis (SDS-PAGE) sample buffer dye and analyzed directly on 8% SDS-PAGE. The protein bands were identified by immunoblotting with α-IGF-1R antibody from Santa Cruz Biotechnology, USA.

### Sub-cellular fractionation

2 × 10^7^ cells of each cell line were harvested and washed thoroughly with 1× PBS. Cells were suspended in lysis buffer (250 mM sucrose, 20 mM HEPES, 10 mM KCl, 1.5 mM MgCl2, 1 mM EDTA, 1 mM EGTA and 1 mM DTT) for 15 min at 4 °C. Cells were centrifuged at 750 × g for 10 min. The nuclear pellet was separated, and supernatant was subjected to ultracentrifugation at 100,000 × g for 1 h. The supernatant was used as cytosolic fraction while pellet was resuspended in lysis buffer containing 0.1% Triton X-100 to obtain plasma membrane fraction. Protein estimation using the Bradford assay was performed before expression analysis of proteins.

### Neuraminidase treatment

*Clostridium perfringes* neuraminidases (Sigma Aldrich, USA) at a concentration of 100 mU/ml were used for desialylation of cell lysates. Briefly, cell lysates from vector control cell lines were incubated with 100 mU of neuraminidase for 3–4 h at 37 °C. Cell lysates from GNE knockdown and vector control were boiled in SDS-PAGE sample buffer and then, subjected to immunoblot analysis.

### Cell cycle analysis

Cells (5 × 10^5^) were seeded in DMEM medium for overnight incubation, followed by 24 h incubation in DCCM medium. After 24 h, the medium was removed, and cells were treated with 5 nM IGF-1 ligand for 10 min. DCCM media was replaced for another 24 h and then, cells were harvested by trypsinization followed by fixation with cold 70% ethanol for 15–20 min on ice. After that, the cells were rinsed with PBS and resuspended in 1 ml of permeabilizing solution containing Triton X-100 (0.05%), RNase A (1 μg/μl, Sigma-Aldrich, USA) and Propidium iodide (100 µg/ml BD Biosciences, USA) and incubated for 15 min at 37 °C. Cell cycle distribution was analyzed using a Flow Cytometer (Beckman Coulter, USA).

### Immunoblotting

Immunoblotting was done on nitrocellulose membrane and PVDF membrane. Antibody dilutions used: Anti-α IGF-1R N-20 1:1000 (Santa Cruz, USA), Anti- β IGF-1R 1:1000 (Santa Cruz, USA), Anti-pIGF-1R 1:1000 (Santa Cruz, USA), Anti-PARP 1:1000 (Santa Cruz, USA), Anti- Procaspase 3 1:1000 (Santa Cruz, USA). Anti-Rabbit-HRP 1: 5000 (Santa Cruz, USA), Anti-Mouse-HRP 1: 5000 (Santa Cruz, USA), Anti-Goat-HRP 1:5000 (Santa Cruz, USA), anti-BAD 1:500 (Santa Cruz, USA), Anti-pBAD 1:500 (Santa Cruz, USA), Anti-ERK 1:1000 (Santa Cruz, USA), Anti pERK 1:1000 (Cell Signaling Technology, USA), Anti AKT 1:1000 (Santa Cruz, USA), Anti pAKT 1:1000 (Cell Signaling Technology, USA), Anti BCL2 1:1000 (Santa Cruz, USA). The blot was developed using ECL (Enhanced Chemiluminescence, Bio-Rad, USA).

### Supplementation with SA

Cells were seeded for 16 h in DMEM media followed by incubation in DCCM media containing 5 mM SA for 24 h.

### IGF-1 treatment

Cells were seeded for 16 h in DMEM media followed by incubation in DCCM media containing 5 nM IGF-1 ligand (Sigma, USA) for 10 min. IGF-1 containing media was removed and washed with PBS followed by cell growth for 24 h in DCCM media.

### Statistical Analysis

All data are expressed as mean ± standard deviation (SD) with three different sets of experiments using GraphPad prism 5 software. One-way analysis of variable (ANOVA) was done followed by Bonferroni posttests, P values for *, ** and *** are <0.05, <0.001 and <0.0001, respectively.

## References

[1] Varki A (2009). Sialic acids in human health and disease. Trends Mol Med..

[2] Hinderlich S, Stäsche R, Zeitler R, Reutter W (1997). A bifunctional enzyme catalyzes the first two steps in N- acetylneuraminic acid biosynthesis of rat liver. Purification and characterization of UDP-N-acetylglucosamine 2-epimerase/N-acetylmannosamine kinase. J. Biol. Chem..

[3] Eisenberg I (2001). The UDP-N-acetylglucosamine 2-epimerase/N-acetylmannosamine kinase gene is mutated in recessive hereditary inclusion body myopathy. Nat. Genet..

[4] Celeste FV (2014). Mutation Update for GNE Gene Variants Associated with GNE Myopathy. Hum. Mutat..

[5] Nishino I (2015). GNE myopathy: current update and future therapy. J. Neurol. Neurosurg. Psychiatry.

[6] Xu X (2017). Safety, pharmacokinetics and sialic acid production after oral administration of N-acetylmannosamine (ManNAc) to subjects with GNE myopathy. Mol. Genet. Metab..

[7] Argov Z (2016). Aceneuramic Acid Extended Release Administration Maintains Upper Limb Muscle Strength in a 48-week Study of Subjects with GNE Myopathy: Results from a Phase 2, Randomized, Controlled Study. J. Neuromuscul. Dis..

[8] Wang Z, Sun Z, Li AV, Yarema KJ (2006). Roles for UDP-GlcNAc 2-epimerase/ManNAc 6-kinase outside of sialic acid biosynthesis: Modulation of sialyltransferase and BiP expression, GM3 and GD3 biosynthesis, proliferation, and apoptosis, and ERK1/2 phosphorylation. J. Biol. Chem..

[9] Amsili S (2008). UDP-N-acetylglucosamine-2-epimerase/N-acetylmannosamine kinase (GNE) binds to alpha-actinin 1: Novel pathways in skeletal muscle?. PLoS One.

[10] Amsili S (2007). Characterization of hereditary inclusion body myopathy myoblasts: possible primary impairment of apoptotic events. Cell Death Differ..

[11] Weidemann W (2006). The collapsin response mediator protein 1 (CRMP-1) and the promyelocytic leukemia zinc finger protein (PLZF) bind to UDP-N-acetylglucosamine 2-epimerase/N-acetylmannosamine kinase (GNE), the key enzyme of sialic acid biosynthesis. FEBS Lett..

[12] Li H (2013). Unfolded Protein Response and Activated Degradative Pathways Regulation in GNE Myopathy. PLoS One.

[13] Fischer C (2013). Cell stress molecules in the skeletal muscle of GNE myopathy. BMC Neurol..

[14] Sela I (2011). The proteomic profile of hereditary inclusion body myopathy. PLoS One.

[15] Grover S, Arya R (2014). Role of UDP-N-Acetylglucosamine2-Epimerase/N-Acetylmannosamine Kinase (GNE) in beta 1-Integrin-Mediated Cell Adhesion. Mol. Neurobiol..

[16] Singh R, Arya R (2016). GNE Myopathy and Cell Apoptosis: A Comparative Mutation Analysis. Mol. Neurobiol..

[17] Chan YM (2017). Substantial deficiency of free sialic acid in muscles of patients with GNE myopathy and in a mouse model. PLoS One.

[18] Sela I (2013). Variable phenotypes of knockin mice carrying the M712T Gne mutation. NeuroMolecular Med..

[19] Niethamer Y (2012). Oral monosaccharide therapies to reverse renal and muscle hyposialylation in a mouse model of GNE myopathy. Mol. Genet. Metab..

[20] Malicdan M (2007). A Gne knockout mouse expressing human GNE D176V mutation develops features similar to distal myopathy with rimmed vacuoles or hereditary inclusion body myopathy. Hum. Mol. Genet..

[21] Huang Z (2014). Inhibition of type I insulin-like growth factor receptor tyrosine kinase by picropodophyllin induces apoptosis and cell cycle arrest in T lymphoblastic leukemia/lymphoma. Leuk. Lymphoma.

[22] Baker J (1993). Role of insulin-like growth factors in embryonic and postnatal growth. Cell.

[23] Liu JP, Baker J, Perkins AS, Robertson EJ, Efstratiadis A (1993). Mice carrying null mutations of the genes encoding insulin-like growth factor I (Igf-1) and type 1 IGFreceptor (Igf1r). Cell.

[24] Velloso C (2008). Regulation of muscle mass by growth hormone and IGF-I. Br. J. Pharmacol..

[25] Cortot AB (2013). Resistance to irreversible EGF receptor tyrosine kinase inhibitors through a multistep mechanism involving the IGF1R pathway. Cancer Res..

[26] Chen C, Xu Y, Song Y (2014). IGF-1 gene-modified muscle-derived stem cells are resistant to oxidative stress via enhanced activation of IGF-1R/PI3K/AKT signaling and secretion of VEGF. Mol. Cell. Biochem..

[27] Humbert S (2002). The IGF-1/Akt pathway is neuroprotective in Huntington’s disease and involves huntingtin phosphorylation by Akt. Dev. Cell.

[28] George C (2017). The Alzheimer’s disease transcriptome mimics the neuroprotective signature of IGF-1 receptor-deficient neurons. Brain.

[29] Nielsen D, Gyllberg H, Ostlund P, Bergman T, Bedecs K (2004). Increased levels of insulin and insulin-like growth factor-1 hybrid receptors and decreased glycosylation of the insulin receptor alpha- and beta-subunits in scrapie-infected neuroblastoma N2a cells. Biochem. J..

[30] Arabkhari M (2010). Desialylation of insulin receptors and IGF-1 receptors by neuraminidase-1 controls the net proliferative response of L6 myoblasts to insulin. Glycobiology.

[31] Thakur S, Garg N, Adamo ML (2013). Deficiency of Insulin-Like Growth Factor-1 Receptor Confers Resistance to Oxidative Stress in C2C12 Myoblasts. PLoS One.

[32] Singh P, Bast F (2015). Screening of multi-targeted natural compounds for receptor tyrosine kinases inhibitors and biological evaluation on cancer cell lines, in silico and *in vitro*. Med. Oncol..

[33] Baserga R, Peruzzi F, Reiss K (2003). The IGF-1 receptor in cancer biology. International Journal of Cancer.

[34] Economou, M. A. Uveal melanoma and muscular degenration:molecular biology and potential therapeutic applications. *Acta ophthalmologica* **86** (2008). 10.1111/j.1755-3768.2008.01188.x 19086934

[35] Kizhakke Madathil S, Evans HN, Saatman KE (2010). Temporal and Regional Changes in IGF-1/IGF-1R Signaling in the Mouse Brain after TraumaticBrain Injury. J. Neurotrauma.

[36] Flynn RS, Murthy KS, Grider JR, Kellum JM (2010). & Kuemmerle, J. F. Endogenous IGF-I and αVβ3 Integrin Ligands Regulate Increased Smooth Muscle Hyperplasia in Stricturing Crohn’s Disease. Gastroenterology.

[37] Valentinis B, Baserga R (2001). IGF-I receptor signalling in transformation and differentiation. Mol. Pathol..

[38] Sadagurski M (2006). Insulin-Like Growth Factor 1 Receptor Signaling Regulates Skin Development and Inhibits Skin Keratinocyte Differentiation. Mol. Cell. Biol..

[39] Peruzzi F (1999). Multiple signaling pathways of the insulin-like growth factor 1 receptor in protection from apoptosis. Mol. Cell. Biol..

[40] Perelman A (2012). JC-1: alternative excitation wavelengths facilitate mitochondrial membrane potential cytometry. Cell Death Dis..

[41] Peters I (1995). CPP32 / Apopain Is a Key Interleukin 1 Converting enzyme like protease involved in Fas-mediated Apoptosis. J. Biol. Chem..

[42] Morales J (2014). Review of Poly (ADP-ribose) Polymerase (PARP) Mechanisms of Action and Rationale for Targeting in Cancer and Other Diseases. Crit. Rev. Eukaryot. Gene Expr..

[43] Simbulan-Rosenthal CM, Rosenthal DS, Iyer S, Boulares H, Smulson ME (1999). Involvement of PARP and poly(ADP-ribo1. Simbulan-Rosenthal, C. M., Rosenthal, D. S., Iyer, S., Boulares, H. & Smulson, M. E. Involvement of PARP and poly(ADP-ribosyl)ation in the early stages of apoptosis and DNA replication. Mol. Cell. Biochem. 193, 137–. Mol. Cell. Biochem..

[44] Chandrasekaran EV (2016). Novel interactions of complex carbohydrates with peanut (PNA), Ricinus communis (RCA-I), Sambucus nigra (SNA-I) and wheat germ (WGA) agglutinins as revealed by the binding specificities of these lectins towards mucin core-2 O-linked and N-linked glycans a. Glycoconj. J..

[45] Robertson DM, Zhu M, Wu YC (2012). Cellular distribution of the IGF-1R in corneal epithelial cells. exp eye res.

[46] Kim JG (2012). Heterodimerization of glycosylated insulin-like growth factor-1 receptors and insulin receptors in cancer cells sensitive to anti-IGF1R antibody. PLoS One.

[47] Lee EK, Gorospe M (2010). Minireview: Posttranscriptional regulation of the insulin and insulin-like growth factor systems. Endocrinology.

[48] Shelton, J. G., Steelman, L. S., White, E. R. & McCubrey, J. A. Synergy between PI3K/Akt and Raf/MEK/ERK pathways in IGF-1R mediated cell cycle progression and prevention of apoptosis in hematopoietic cells. *Cell Cycle* **3** (2004). 14726697

[49] Song G, Ouyang G, Bao S (2005). The activation of Akt/PKB signaling pathway and cell survival. J Cell Mol Med.

[50] Garwood CJ (2015). Insulin and IGF1 signalling pathways in human astrocytes *in vitro* and *in vivo*; characterisation, subcellular localisation and modulation of the receptors. Mol. Brain.

[51] Schönthal AH, Pozarowski P, Darzynkiewicz Z (2004). Analysis of Cell Cycle by Flow Cytometry 301 301 Analysis of Cell Cycle by Flow Cytometry. Checkp. Control. Cancer.

[52] Bosch-Morató M (2016). Increased amyloid β-peptide uptake in skeletal muscle is induced by hyposialylation and may account for apoptosis in GNE myopathy. Oncotarget.

[53] Eisenberg I (2008). Mitochondrial processes are impaired in hereditary inclusion body myopathy. Hum. Mol. Genet..

[54] Itkonen HM, Mills IG (2013). N-Linked Glycosylation Supports Cross-Talk between Receptor Tyrosine Kinases and Androgen Receptor. PLoS One.

[55] All-Ericsson C (2002). Insulin-like growth factor-1 receptor in uveal melanoma: a predictor for metastatic disease and a potential therapeutic target. Investig. Ophthalmol. {&} Vis. Sci..

[56] Dricu A (1999). Expression of the insulin-like growth factor 1receptor (IGF-1R) in breast cancer cells: Evidence for a regulatory role of dolichyl phosphate in the transition from an intracellular to an extracellular IGF-1 pathway. Glycobiology.

[57] Sarfstein R, Werner H (2013). Minireview: Nuclear insulin and insulin-like growth factor-1 receptors: A novel paradigm in signal transduction. Endocrinology.

[58] Warsito D, Sjöström S, Andersson S, Larsson O, Sehat B (2012). Nuclear IGF1R is a transcriptional co-activator of LEF1/TCF. EMBO Rep..

[59] Sun H (2003). Insulin-like growth factor I receptor signaling and nuclear translocation of insulin receptor substrates 1 and 2. Mol. Endocrinol..

[60] Zhang Y, Liu R, Ni M, Gill P, Lee AS (2010). Cell Surface Relocalization of the Endoplasmic Reticulum. J. Biol. Chem..

[61] Yen H (2015). Effect of sialylation on EGFR phosphorylation and resistance to tyrosine kinase inhibition. PNAS.

[62] Harazi A (2014). Survival-apoptosis associated signaling in GNE myopathy-cultured myoblasts. J. Recept. Signal Transduct..

[63] Sayeed A, Alam N, Trerotola M, Languino LR (2012). Insulin-like growth factor 1 stimulation of androgen receptor activity requires β1A integrins. J Cell Physiol..

[64] Sakowski SA, Schuyler AD, Feldman EL (2009). Insulin-like growth factor-I for the treatment of amyotrophic lateral sclerosis. Amyotroph Lateral Scler..

[65] Russo VC, Gluckman PD, Feldman EL, Werther GA (2005). The Insulin-Like Growth Factor System and Its Pleiotropic Functions in Brain. Endocr. Rev..

[66] Conti E (2008). Recombinant human insulin-like growth factor-1: a new cardiovascular disease treatment option?. Cardiovasc. Hematol. Agents Med. Chem..

[67] Palazzolo I (2009). Overexpression of IGF-1 in Muscle Attenuates Disease in a Mouse Model of Spinal and Bulbar Muscular Atrophy. Neuron.

